# Suicide Mortality in Foreign Residents of Japan

**DOI:** 10.3390/ijerph16173013

**Published:** 2019-08-21

**Authors:** Stuart Gilmour, Haruko Hoshino, Bibha Dhungel

**Affiliations:** Graduate School of Public Health, St. Luke’s Center for Clinical Academia, Susumu & Mieko Memorial, St. Luke’s International University, 3-6-2 Tsukiji, Chuo-ku, Tokyo 104-0045, Japan

**Keywords:** suicide, mortality, epidemiology, Japan, migrant health

## Abstract

Suicide is a major public health issue in Japan, with very high rates of death compared to other countries in the Asia Pacific. Foreigners living in Japan may be at increased risk of suicide, but little is known about how their risk of suicide differs from that of their country of origin or Japanese nationals. We used data on suicide mortality from the Japan Vital Registration System for the period 2012–2016 to analyze risk of suicide mortality in Japan for Japanese, Korean, Chinese, and other nationalities living in Japan, adjusting for age and separately by sex. We estimated standardized mortality rates using both the Japanese population as a reference, and also the population of the home nation of the foreign residents. We found that Korean nationals living in Japan have significantly higher mortality rates than Japanese nationals, and that the suicide mortality rate of Korean nationals living in Japan is higher than in their home country, but that this is not the case for Chinese or other nationals resident in Japan. Koreans living in Japan have a very high risk of mortality due to suicide which may reflect the special social, economic, and cultural pressures they face as a marginalized population in Japan.

## 1. Introduction

Suicide remains a major global cause of mortality [1]. Suicide rates in Japan are high, and suicide remains one of the ten highest causes of death in Japan [2]. Although suicide rates have been declining over time [3], there are significant variations in patterns of suicide mortality by occupation [4], and rates remain particularly high among working-age men [5]. Workplace stress [6] and economic change [7,8] have been suggested as particularly important causes of the high rate of suicide in Japan, along with particular social and cultural factors that may differ from other nations in Asia, such as patterns of group suicide [9] and particular cultural attitudes towards suicide [10]. 

The role of workplace stress in suicide in Japan and the high prevalence of suicide mortality among working-age professional and managerial roles potentially raises the risk of suicide among non-Japanese who migrate to Japan. In the past 10 years, the number of foreigners entering Japan for work has increased, reaching the highest number ever in 2017 [11], and the government continues to ease visa restrictions to increase the number of long-term migrants entering Japan [12]. As the number of migrants moving to Japan to work continues to increase, and more migrants settle for longer in Japan, their risk of suicide may increase relative to their home countries, and the Japanese health system may begin to face an increasing burden of suicide-related care for these people. Currently, however, little is known about the suicide risk facing foreigners in Japan. Foreigners in Japan face particular challenges to the management of their physical and psychological health, in particular due to cultural differences and language barriers [13], but also challenges due to marginalization, racism, and exclusion, which can be alleviated through social networks and psychosocial support [14]. In this context, research about the rate of suicide mortality among foreigners in Japan, and comparison with rates in the home country of foreign residents, is important to understand how suicide risk differs in foreigners living in Japan. One recent study calculated age-standardized suicide rates in Japan and compared them with age-standardized rates in the home country of the non-Japanese nationals living in Japan [15], but this comparison was incomplete because the study did not standardize the mortality rate of the deaths in Japan relative to those in the foreign residents’ home countries. Since migrant populations in Japan are likely to be younger, working, and potentially have a different gender distribution to the populations of their home countries, it is important to standardize mortality rates in these populations in Japan against those of their home country in order to properly understand the risks they face.

This study uses data on the most recent five years of mortality data in Japan to:

- Estimate age-standardized suicide mortality rates among major nationalities of foreigners living in Japan and compare them to age-standardized suicide mortality rates of Japanese nationals;
- Estimate age-standardized suicide mortality rates for foreigners in Japan using the population of their home nation as reference, to compare the suicide risk of foreigners living in Japan with the risk they would have experienced in their home country;
- Describe patterns in age- and sex-specific mortality among foreigners living in Japan using a comprehensive Poisson loglinear regression model.

This study provides improved insight into whether foreigners in Japan retain the suicide rate of their home country, converge to the Japanese suicide rate, or experience a new mortality pattern unlike that of their home or their host country. This information will help to understand whether suicide mortality presents a social contagion risk to people migrating to countries with high suicide rates and also help to identify potential strategies to reduce suicide mortality in Japan.

## 2. Materials and Methods 

We obtained data on causes of death from the vital registration system of Japan for the period 2012–2016. These data are collected by the Ministry of Health, Labor, and Welfare (MHLW) of Japan and cover all deaths that occur for any cause, including non-Japanese nationals who die in Japan. Suicide was defined as any death with an external cause of death coded using ICD-10 codes of X60-69, X70-75, and X80-84. Age in five-year age categories (0–4, 5–9, up to 85+), sex, and year of death were also obtained. Because the number of foreigners living in Japan is small and the number of events was expected to be small, we aggregated data for the five-year period, so that we calculated the total number of deaths over five years. We then divided by the population in the 2015 census and multiplied by 20,000 to obtain an average suicide rate per 100,000 over the five years. Nationality of all deaths was obtained and divided into four categories: Japanese, Korean, Chinese, and other. Other primarily includes Brazilian, Philippine, and other south East Asian nationalities, but even aggregating over five years, the number of deaths in these groups is too small to disaggregate. Mortality rates were calculated separately for men and women.

Mortality rates for all four nationalities were standardized to the Japanese population (foreign-born and local) from the 2015 census. This made almost no difference to the mortality rate for Japanese nationals but slightly changed rates for non-Japanese nationals with a different population structure (in Japan) than the Japanese population. We also standardized the mortality rate for foreigners to the 2015 reference population for their home country, using the global population for the “other” nationality category. This was done because it is possible that the population distribution of foreigners living in Japan is quite different to that of their home country and could be expected to have a different suicide mortality rate due to this difference in population structure. Population data for China, Korea, and the global population were obtained from WHO sources. These directly standardized rates were compared to estimated directly standardized suicide mortality rates in the home country of these nationals, obtained from the global burden of disease project [1]. 

Finally, a Poisson regression model was constructed separately by age and sex to estimate the relative risk of suicide mortality in Korean, Chinese, and other nationalities living in Japan compared to Japanese nationals, after adjusting for age. All analyses were conducted in Stata/IC 15 (StataCorp, College Station, Texas, USA).

## 3. Results

The crude suicide rate in Japanese men over the period studied was 27.9 per 100,000, and in Japanese women it was 11.5 per 100,000. Table 1 shows the crude suicide rate for Korean nationals, Chinese nationals, and all other nationalities in Japan, by sex, the directly standardized rate with a Japanese reference population, and the directly standardized rate with their respective home countries as a reference population. For other nationalities, the global population was used as reference population. From Table 1, it is clear that suicide mortality among Korean nationals living in Japan is higher than it would be in their home country. The suicide rate for Chinese nationals living in Japan is the same as it would be if they were living in China, while the suicide rate among other non-Japanese nationals living in Japan is lower than it would be in their home countries. This pattern can be observed for men and women. It is also clear from Table 1 that after standardization to the age structure of the Japanese population, Korean suicide rates remain higher than those of the Japanese population, while Chinese and other nationalities are lower than those of the Japanese population. 

**Figure 1:**
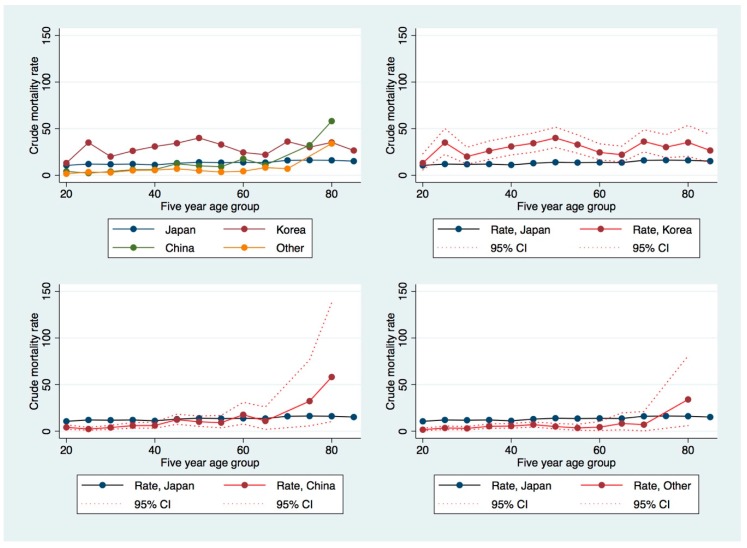
Age-specific crude suicide mortality rates by nationality for women. Clockwise from top left: age-specific rates for Japan, China, Korea, and all other countries; age-specific rates for Korea, all other nations, and China, respectively, with 95% confidence interval and Japanese mortality rates in blue as reference.

Figure 1 shows the age-specific suicide mortality rate for female Japanese, Korean, Chinese, and other nationals in Japan, with confidence intervals. Suicide rates are higher in almost all age groups for Koreans and are lower for Chinese aged below 45, or for all other nationalities aged below 65. For older non-Korean age groups, uncertainty is very high. This figure suggests that the risk of suicide among Chinese and other nationalities in Japan rises with age and is similar to that of Japanese women at older age groups, indicating the need for focused antisuicide measures amongst the eldest non-Japanese nationals living in Japan.

**Figure 2:**
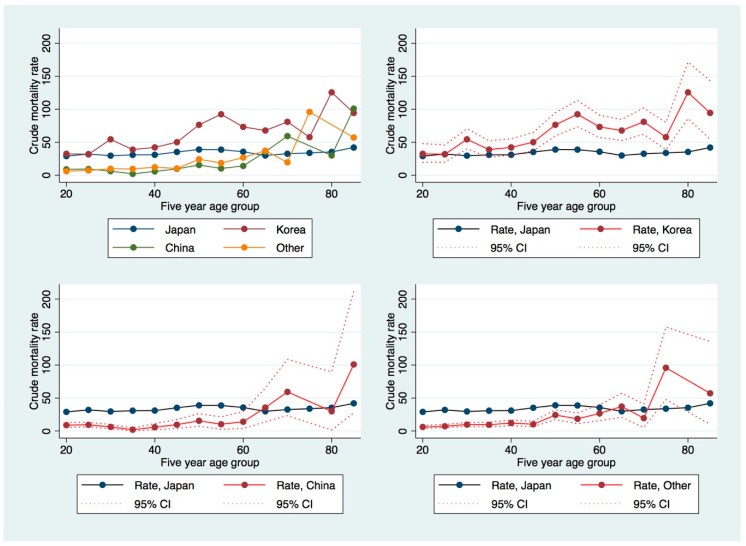
Age-specific crude suicide mortality rates by nationality for women. Clockwise from top left: age-specific rates for Japan, China, Korea, and all other countries; age-specific rates for Korea, all other nations, and China, respectively, with 95% confidence interval and Japanese mortality rates in blue as reference.

Figure 2 shows the age-specific suicide mortality rate for male Japanese, Korean, Chinese, and other nationals in Japan, with confidence intervals. Suicide rates are higher in almost all age groups for Koreans and are lower for Chinese and other nationalities aged below 60. For older non-Korean age groups, uncertainty is very high. Older men of all non-Japanese nationalities have extremely high suicide rates.

The full results of the loglinear regression model of suicide mortality are shown in Table 2, separately for men and women. Note that the model coefficients are exponentiated in this table and presented as rate ratios, and age has been modified so that the intercept gives the mortality rate in 15-year-olds, averaged over the five-year period of the data. Thus, the coefficient of age shows the (multiplicative) increase in mortality due to a one-year increase in age, relative to the mortality rate in 15-year-olds. The model confirms that suicide rates are higher in Koreans, and that there are very different age patterns between Japanese and non-Japanese nationals in Japan. Korean women have a significantly higher baseline suicide risk but a similar age trend to Japanese women, but all other nationalities show a different age pattern for both men and women.

The interaction terms in Table 2 are difficult to interpret as presented, so in Table 3, the interaction terms are combined to show the specific age patterns of mortality for each nationality, separately by sex. The coefficients presented here show the percentage change with a single year’s increase of age for a person of the given nationality and sex, relative to a 15-year-old of the given nationality and sex. Thus, for example, for a Chinese man, every year of age is associated with a 3.27% increase in the mortality rate due to suicide. Korean women see no increase in suicide mortality risk with age, but from Table 2, their suicide rate is 2.46 times higher than that of Japanese women.

## 4. Discussion

This research used mortality data for the period 2012–2016 to compare suicide mortality rates for Japanese nationals and foreigners resident in Japan. We calculated average crude mortality rates over the five-year period and also standardized mortality rates using both the 2015 Japan population and the 2015 population of the origin country of nationals living in Japan. This calculation enabled us to show both how mortality rates for foreigners compare to the Japanese population after adjusting for their different age distribution and how mortality rates compare to their home country, after adjusting for the likely differences in age structure of nationals of those countries who have migrated to Japan. 

We found that suicide mortality among Korean nationals living in Japan is significantly higher than that of Japanese nationals, even after adjusting for age, and that the mortality rate for these nationals is also significantly higher than it would be if they had remained in Korea. By contrast, Chinese nationals living in Japan have lower suicide mortality rates than Japanese after adjusting for age, and their suicide mortality rate is not significantly different than that of their home country. Nationals of other countries in Japan have lower suicide mortality rates than Japanese nationals, and their suicide mortality rate is also lower than the global rate after standardization. These patterns held for men and women and suggest that suicide mortality in Koreans resident in Japan is significantly elevated relative to their home country, while there is no change in Chinese, and lower rates in foreigners from other nations. The increase in standardized suicide mortality rates for Koreans was large, rising from 36.3 to 46.3 per 100,000 in men and from 15.5 to 24.1 per 100,000 in women. After adjustment for age in a Poisson regression model, Korean women had more than twice the risk of suicide mortality as Japanese women, and this risk was approximately constant across the entire adult age range.

The reasons for this increase in suicide risk among Koreans living in Japan are not clear. Previous comparisons of suicide risk in Japan and Korea have found a rapidly increasing suicide rate among Korean women and changing gender ratios in the suicide patterns of Koreans [16], but limited explanation for why this pattern of suicide might be changing. A previous study suggested that higher suicide rates among Koreans may be partially a consequence of discrimination [17]. Koreans living in Japan experience a unique history of discrimination and marginalization connected to the forced residence of many Koreans in Japan after the colonial period in the first half of the 20th century, and special Japanese cultural and political attitudes towards this population (commonly referred to as *zainichi* Koreans) in the Japanese polity [18]. Koreans are unique among all foreigners living in Japan in that a significant portion of the total population of Koreans living in Japan were born in Japan. Data from the Ministry of Justice in 2018 shows that 66% of Koreans living in Japan are in the *special permanent resident* visa category, compared to just 0.1% of Chinese. This visa is granted to foreigners with ancestry related to Japan’s former colonies, and the very high proportion of Koreans with this visa class reflects the special historical situation of *zainichi* Koreans in Japan. Given that 66% of Korean residents in Japan are *zainichi*, it is likely that most of the suicides recorded as being “Korean” in the ministry of health vital registration data actually are deaths of 2nd-, 3rd- or 4th-generation Koreans who have been unable to adopt Japanese citizenship, and not migrants from their home country living and working temporarily in Japan; this special status of many Koreans may be a particular source of stress and mental illness compared to other nationalities living in Japan, with consequences for suicide risk in this population. Their situation stands in stark contrast to the mortality rates of other nationalities in Japan, which appear to be reduced relative to the global average, possibly because of the benefits of moving to a comparatively wealthier country with universal health coverage (UHC).

The data used in our study do not provide information on the length of time that the individuals were settled in Japan, so it is not possible to draw any conclusions about the time between arrival in Japan and suicide mortality. This information could be important for Chinese and nationals of other countries in particular, since the vast majority of these groups were adult migrants to Japan, and the length of time between arrival and suicide death is relevant to making policy about suicide prevention for these groups. More research on the detailed life trajectory of adult foreigners in Japan who commit suicide after migration is needed to inform better suicide prevention policy for migrants in Japan. Since it is known that psychiatric problems and mental health are strongly associated with suicide risk, it is important to conduct further research on the mental health issues of new migrants to Japan, and also their ability to access appropriate psychiatric and psychological care while in Japan. Conversely, there is some evidence of a healthy migrant effect in some migrants coming to Japan, though it is not clear this applies to suicide specifically [19]. This effect could also not be explored in this study, and without detailed personal information in a high-quality study focused on mental health and suicide risk, it remains unclear to what extent migrants from China or other countries have self-selected for better health.

Koreans and Chinese are the largest two migrant populations in Japan and constitute the bulk of non-Japanese suicide deaths in this study. It is noteworthy that their suicide mortality rates have increased or not changed relative to their home countries, even though they are living in a country that is world-renowned for having very good health outcomes [20]. This may partially reflect the stresses related to migration and adaptation, but it may also arise from shortcomings in Japan’s suicide prevention policies. Since 2006, Japan has had a strong suicide prevention policy framework [21,22], age-specific suicide rates have been falling consistently over the past 20 years [3], as have rates of self-harm [23], but the *Basic Act for Suicide Prevention* does not specify any particular activities or interventions targeted at marginalized groups in Japan, including migrants. The countermeasures described in the act are extensive and based on evidence but include a wide range of activities focused around reporting of risk behavior and engagement of community social capital, which may be difficult for marginalized groups to benefit from. In particular for migrants, who often lack language skills and may not have the same rights to employment and housing security as Japanese nationals, the focus of current Japanese suicide prevention activities on the engagement of social capital and community support for suicide prevention may not be as effective. Given the high rate of suicide mortality particularly among Korean nationals living in Japan and the limited change observed in Chinese nationals, the government should consider expanding and enhancing the current suicide countermeasures in place in Japan to include a specific focus on migrants and non-Japanese, and additional effort to adapt suicide prevention policy to cover marginalized groups in Japan, including but not limited to foreign residents. Furthermore, the extremely high rate of suicide in Koreans living in Japan needs to be recognized and additional efforts made to counter discrimination against and marginalization of Koreans living in Japan. This should include renewed efforts to normalize the citizenship status of multigenerational *zainichi* Koreans and further efforts to reduce discrimination and end hate speech. Detailed investigation is also needed of the reasons for the high rate of suicide amongst Koreans resident in Japan, and a better understanding of the particular factors that put them at risk.

## 5. Conclusions

Suicide rates among non-Japanese nationals in Japan vary significantly by the nation of origin of those nationals and are particularly high among Koreans living in Japan. While people of many nationalities have lower suicide rates in Japan than in their country of origin, Chinese suicide mortality rates do not change relative to mainland China, and the suicide rates of Koreans living in Japan are significantly higher than those of both Japanese nationals and Koreans living in Korea. This may reflect the consequences of discrimination and marginalization. As the number of foreign residents in Japan increases and the Japanese government expands its migration targets to draw in more migrants and low-skilled employees from around Asia, the government needs to update and adapt its suicide prevention policies to include particular responses aimed at marginalized groups and at foreigners living in Japan who may lack the social capital, language skills, and rights required to fully benefit from suicide prevention activities. By expanding and revising suicide prevention policies to respect the particular risks experienced by marginalized people and to explicitly include foreign residents of Japan in its countermeasures, the Government of Japan can improve the health of foreigners living in Japan and ensure that as Japan opens to the world, it also becomes a more inclusive and equitable society that guarantees health for all of its population.

## Figures and Tables

**Table 1:** Age-standardized suicide mortality rates by sex and nationality.

Sex and Nationality	Crude Rate	Directly Standardized Mortality Rate (Japanese Population)	Reference Mortality Rate (Japan)	Directly Standardized Mortality Rate (Home Population)	Reference Mortality Rate (Home Nation^1^)
Male					
Japan	27.9 (27.8–28.1)	27.9 (27.8–28.1)	27.9	27.9 (27.8–28.1)	11.5
Korea	55.3 (51.1–59.5)	51.3 (46.6–56.0)	27.9	46.3 (41.8–50.7)	36.3
China	8.0 (6.6–9.5)	15.0 (9.6–20.4)	27.9	9.7. (7.1–12.3)	10.7
Other	10.3 (9.1–11.4)	18.3 (13.9–22.8)	27.9	10.8 (9.1–12.5)	15.6
Female					
Japan	11.5 (11.4–11.7)	11.5 (11.4–11.7)	11.5	11.5 (11.4–11.7)	11.5
Korea	27.4 (24.8–30.1)	24.7 (21.7–27.7)	11.5	24.1 (21.1–27.0)	15.5
China	5.7 (4.7–6.7)	10.0 (5.2–14.7)	11.5	7.1 (5.2–9.1)	6.5
Other	4.1 (3.4–4.8)	5.4 (2.8–8.0)	11.5	3.8 (2.7–4.8)	7.0

^1^ Source: Global Burden of Disease Study [1].

**Table 2:** Poisson regression model of suicide mortality risk by sex.

Variable	Rate Ratio	95% Confidence Interval	Z Statistic	*p* value
Male model				
Intercept	0.00130	0.01286–0.00132	−918.55	<0.001
Age	1.0061	1.0058–1.0065	34.05	<0.001
Nationality				
Japanese	1			
Korean	1.1932	0.9598–1.4833	1.59	0.1
Chinese	0.1810	0.1229–0.2667	−8.65	<0.001
Other	0.1929	0.1480–0.2516	−12.15	<0.001
Nationality/age interaction				
Japanese	1			
Korean	1.0121	1.0069–1.0173	4.56	<0.001
Chinese	1.0264	1.0129–1.0401	3.85	<0.001
Other	1.0315	1.0259–1.0405	7.01	<0.001
Female model				
Intercept	0.00048	0.00047–0.00049	−664.83	<0.001
Age	1.0084	1.0078–1.0089	31.98	<0.001
Nationality				
Japanese	1			
Korean	2.4650	1.8851–3.2207	6.60	<0.001
Chinese	0.2555	0.1652–0.3974	−6.05	<0.001
Other	0.2503	0.1593–0.3935	−6.00	<0.001
Nationality/age interaction				
Japanese	1			
Korean	0.9974	0.9909–1.0039	−0.79	0.4
Chinese	1.0338	1.0187–1.0491	4.43	<0.001
Other	1.0187	1.0026–1.0350	2.28	0.02

**Table 3:** Percentage change in mortality for a year’s increase in age by sex and nationality.

Nationality	Percentage Change	95% Confidence Interval	Z Statistic	*p* value
Male model				
Japanese	0.61	0.58–0.65	34.05	<0.001
Korean	1.83	1.31–2.35	6.89	<0.001
Chinese	3.27	1.91–4.65	4.76	<0.001
Other	3.78	2.89–4.68	8.4	<0.001
Female model				
Japanese	0.84	0.78–0.89	31.98	<0.001
Korean	0.57	−0.09–1.22	1.72	0.09
Chinese	4.24	2.72–5.79	5.54	<0.001
Other	2.72	1.10–4.38	3.30	0.001
